# Same storm, different boats: the impact of COVID-19 on Black students and academic staff in UK and US higher education

**DOI:** 10.1007/s10734-022-00939-0

**Published:** 2022-10-25

**Authors:** Jason Arday, Christopher Jones

**Affiliations:** 1 School of Education, St Andrew’s Building, University of Glasgow, 11 Eldon Street, Glasgow, G3 6NH UK; 2 Department of Sociology, Higher Education and Social Inequalities, Durham University, 32 Old Elvet, Durham, DH1 3HN UK

**Keywords:** COVID-19, Pandemic, Education, Race and racism, Higher education, Mental health, Health inequality

## Abstract

The permanence of systemic racism in the UK and USA means that Black people are disadvantaged in myriad ways, including within the Academy. While the disproportionate impact of COVID-19, alongside the Black Lives Matter movement, has increased awareness of the challenges faced by Black communities, these issues remain, both in and beyond higher education. Furthermore, there is still a paucity of research individualising the experiences of Black people, who are often homogenised with other ethnic minority groups. This paper explores the impact of COVID-19 on UK and US Black students and academic staff, utilising a critical race theory (CRT) framework. Analysis revealed that Black students and staff experienced COVID-19 against the backdrop of racism as a “pandemic within a pandemic” (Laurencin and Walker, Cell Systems 11:9–10, 2020), including racial (re)traumatisation, loneliness and isolation. Other themes included precarious employment and exploitation. Recommendations are offered for penetrative interventions that can support Black students and staff in the wake of strained race relations neglecting their adverse experiences and a global pandemic.

## Introduction

“We are in the same storm, but we are not all in the same boat”

Barr (2020), cited in Zhou and Kan (2021)

The arrival of COVID-19 brought with it unprecedented global change. At the time of writing, the USA had almost 47 million cases (World Health Organisation, 2021), and Britain was on its way to 10 million (UK Government, 2021), with numbers rising as the winter months approached. The most effective strategy to minimise transmission of the virus has been to restrict movement of people, including quarantine, isolation (e.g. Webster et al., 2020) and a series of national lockdowns in the UK (Zhou and Kan, 2021) . In a global first, this has included the closure of schools and universities, who instead opened their virtual doors to students for much of 2020. Though the vaccine has enabled greater movement, including the return of face-to-face higher education, the pandemic, associated restrictions and their collective impact persist. Those with direct experience of COVID-19 are more likely to be depressed, anxious (Rogers, 2021) and experience post-traumatic stress disorder (Chamberlain et al., 2021), while as a wider group, feelings of stress and loneliness are at their highest in over a decade (Li & Wang, 2020; Mental Health Foundation, 2021).

The title of this paper highlights how different ethnic groups within the USA and UK are not all in the same boat but actually, *all in the same storm*. As Damian Barr explains (2020) while ‘some of us are on super-yachts, some have just the one oar’. When a storm like COVID-19 hits, it became visible that people racialised as Black are some of the most vulnerable in society; caught in the eye of the storm with very few material tools to survive it. While the impact of COVID-19 has been far-reaching, evidence points to specific groups being at greater risk of its effects. In terms of mental health impacts, for example, those at highest risk are young adults (e.g. Public Health England, 2021), women (e.g. Niedzwiedz et al., 2021), ethnic minority communities (e.g. Proto & Quintana-Domeque, 2021), people experiencing socioeconomic adversity (e.g. Wilson, 2021) , unpaid carers (e.g. O’Shea, 2021) and clinically vulnerable groups such as those with disabilities (e.g. Pierce et al., 2021). The Doreen Lawrence review captures the systemic nature of the problem, recognising that “Covid-19 has thrived on inequalities that have long scarred British society” (2020, p. 24).

### The impact of COVID-19 on Black, Asian and ethnic minority communities

The pandemic has further illuminated the wide-reaching consequences of structural racism: Black men are 4.2 times more likely to die from a COVID-19-related death than White men (Office for National Statistics, 2020a), while the risk of infection is 56% higher within Black, Asian and ethnic minority communities compared to White British (Kenway & Butt, 2020). The first ten healthcare professionals in the UK to die from COVID-19 were from the Black, Asian and ethnic minority communities (Phiri et al., 2021), as were one-third of the COVID-19 patients admitted to critical care units in 2020—of which the highest percentage (14%) were Black (Intensive Care National Audit Research Centre [ICNARC], 2020). Accompanying the aggregate of research in the UK (e.g. Public Health England, 2020a; Razaq et al., 2020; Williamson et al., 2020) , a similar pattern is observed in the USA (e.g. Killerby et al., 2020; Lo et al., 2021), with death rates for Indigenous (3.3 ×), Pacific Islander (2.6 ×), Latinx (2.4 ×) and Black (2 ×) higher than White and Asian Americans (APM Research Lab, 2021) and rates of hospitalisation higher across ethnic minority communities than White (Buikeme et al., 2021) .

With each COVID-19 death leaving behind bereaved family members (Simon et al., 2020), there is a predicted surge in post-pandemic mental health disorders disproportionately affecting ethnic minorities (Thomas, 2021). The current mental health impacts of COVID-19 on ethnic minorities are notable (e.g. Smith et al., 2020), with ethnic minorities in the USA reporting disproportionately worse mental health outcomes (Czeisler et al., 2020) and in the UK reporting higher levels of depression, anxiety, abuse, self-harm and thoughts of suicide since before the pandemic (Iob et al., 2020; Pierce et al., 2020). Findings from Proto and Quintana-Domeque indicate that Bangladeshi and Indian men experienced a higher risk of mental health problems than White men (2021), though research from the UK Household Longitudinal Study found no interethnic differences in mental health problems when comparing pre- and during-pandemic data (Daly et al., 2020). Other studies suggest that it is unlikely ethnicity itself causes differences in mental health outcomes, but rather correlations with other factors such as occupation, low income, higher likelihood of infection or death and racism (e.g. John et al., 2020; Nazroo et al., 2020).

The clear and disproportionate effect of COVID-19 on ethnic minorities across the USA and UK thus highlights the impact of racism on physical and mental health. As Razai et al. (2021) , racism both shapes social determinants of health and has its own effect on the health of ethnic minorities. These social determinants include socioeconomic status, living in urban areas, poor and overcrowded housing, high risk occupations, comorbidities and cultural barriers, all of which ethnic minority groups are more likely to be adversely impacted by Razai et al. (2021). Over time these have, and continue to, lead to worse health outcomes for minoritised communities—as J. Kēhaulani Kauanui states, ‘racism is a structure, not an event’ (2016). It is unsurprising, then, that Public Health England (2020b) have suggested racism and discrimination may have contributed to increased risk of exposure to and death from COVID-19 among ethnic minority groups and that discrimination, cultural and institutional racism are empirically associated with ethnic disparities in COVID-19 outcomes (Razai et al., 2021). ‘White supremacy’, much like COVID-19, is a virus (Menakem, 2021). When speaking about the impact of COVID-19 on ethnic minorities, therefore, we must never stray far from the topic of racism.

### Black Lives Matter

While the purpose of this article was not to explain why *Black Lives Matter*,^1^ it has done so by virtue of the data. As the evidence reflects, Black people are among the most disproportionately impacted by COVID-19 both across the USA (e.g. APM Research Lab, 2021) and UK (e.g. Office for National Statistics, 2020a) contexts. Considering the current and historic experiences of Black people in a multitude of areas such as housing (Joseph Rowntree Foundation, 2021), employment (House of Commons Library (2018)) , income (Department for Work Pensions, 2021), poverty rates (Social Metrics Commission, 2020), criminal justice (Lammy, 2017) and health (Institute of Health Equity, 2020), the impact of COVID-19 on the Black community is devastating but unsurprising. And yet, it was the public murder of George Floyd by Minnesotan police that marked a ‘rest-of-the-world’ awakening to the lived reality of Black communities. By summer 2020, the Black Lives Matter movement rose to global prominence which, whilst reigniting a collective desire to fight for Black human rights, also ‘reopened psychosocial wounds’ (Crooks et al., 2020). Together with arrival of COVID-19, itself considered a traumatic event (Ettman et al., 2020) , for Black communities, 2020 and 2021 have been a ‘pandemic within a pandemic’ (Laurencin & Walker, 2020). The ongoing (re)traumatisation of Black communities thus persists, and with it a need to tend to and centre Black mental health. This is echoed in the evidence base, which calls for more attention to be given to Black lives (Thomas, 2021), particularly Black women (Pennant, 2022) , and especially Black women in academia (Walton et al., 2021) who are centred in the current study.

### Black in academia

One of the hotspots where inequality thrives is the Academy, which has long been associated with structural racism (e.g. Arday & Mirza, 2018; Pennant, 2022) . Black students experience greater mental health problems and difficulties transitioning into the Academy (Arday, 2020), having navigated a schooling system with a ‘whitewashed curriculum’ (Dowling & Flintoff, 2015), disproportionate rates of exclusions (Timpson, 2019) and anti-Black racism (YMCA, 2020). For example, Bird and Pitman (2020) examined the lack of diversity on reading lists at two research intensive UK universities and found only 7% of the Social Sciences authors reviewed were from “BAME” backgrounds—despite there being a 39% UK domiciled student population. Also, in Mwangi et al. (2018) study ‘Black elephant in the room’, *Black students contextualising campus racial climate within US racial climate*, Black student participants ‘demonstrated how the systemic anti-Black racism being resisted through larger movements such as Black Lives Matter is also reflected and reproduced in US universities’ (p. 469). The effects of these show anti-Black racism is fluid and relentless (Gillborn, 2018), reflected in attainment and outcomes of young Black students at key educational stages including school (Department for Education [UK], 2021) and university (Universities UK, 2019). Among Black staff, institutional racism yields different but equally immobilising effects, such as higher numbers of precarious contracts (UCU, 2020), lower retention rates (EHRC, 2019) and restrictions to development and progression (Arday & Mirza, 2018). As Bowden and Buie (2021) note, the way these manifest are often covert and insidious:

One does not need explicitly racist policies to yield racial disparities. For example, providing grant supplements only to those with active awards is not openly racist; however, when you consider that Black principal investigators are funded at a much lower rate than their white colleagues, the result is less access for Black researchers to these funding opportunities. (p. 760)

These challenges are reflected in the mental health experiences of Black students (Arday, 2018) and staff (Arday, 2021), perpetuated by stigmatisation, lack of belonging, isolation and marginalisation. For Black women in particular, the lack of support beyond that which is self- and communally cultivated creates pressures that can lead to mental health implications (Pennant, 2021). With staff in particular, these pressures are exacerbated when their White female counterparts are active participators in their exclusion in the workplace, and thus strategies are taken for self-care and survival (Rollock, 2019). Compounding these experiences are the differential healthcare outcomes for Black staff and students who do attempt to access support, which include overdiagnosis (Myrie & Gannon, 2013) and undertreatment (Evans-Lacko et al., 2014), as well as a lack of culturally responsive services (Arday, 2018).

Unsurprisingly when considering the evidence, the sudden imposition of online teaching, cancellation of lectures, use of alternative assessments and adjustments to new technology have had differential impacts on students and staff of colour. Research shows that over the pandemic, ethnic minority groups, including those from the Black community, have had reduced access to research resources and have been more adversely affected in terms of mental wellbeing, social isolation and academic support (Goldstone & Zhang, 2021). Undoubtedly, there are also incoming Black university students impacted by the use of algorithms and teacher assessments, which together produced disproportionate downgrading of GCSE and A-Level results^2^ (Murphy & Wyness, 2020). In the USA, there are higher levels of attrition in higher education among ethnic minorities (Aucejo et al., 2020) and steep declines in university and college enrolment since the pandemic began, with a 14.3% drop among Black men (Department of Education [US], 2021) . Some of the largest barriers reported were acclimation to the online curriculum, financial constraints and prioritising care-giving responsibilities; though without disaggregation of ethnic minority data, it is unclear whether these experiences represented those of Black students. Similarly in the UK, while young carers are 1.5 times as likely to be from Black, Asian and minority ethnic communities (The Children’s Society, 2020), it is not clear what proportion of these are Black young people. This exemplifies, and further reinforces, the need to disaggregate ethnic minority data if we are to better understand the impact of COVID-19 on Black students. As Pennant notes, there is a need to support Black students, especially Black girls and women, who continue to endure the sharper end of both racism and the pandemic (2021, p. 13). For instance, prior to the pandemic, young Black girls have been rendered invisible in secondary education regarding academic success (e.g. high exclusion rates similar to boys, see Rollock, 2007). Therefore, the racialisation of gender is multi-dimensional and important to consider amongst young Black pupils in the academy.

Meanwhile, university staff are feeling the weight of responsibility to manage the impact COVID-19 has had on their students—and in particular, Black academic staff who do so with an additional ‘Black Tax’^3^ (Walton et al., 2021). Black women bear the weight of this even more so, describing the pressure to ‘save our students from the stress and weight of the pandemic’ and ‘save our departments from the increased scrutiny around how we view, assess and address diversity, equity and inclusion’ (Walton et al., 2021, p. 252), particularly in line with the concurrent upsurge of the Black Lives Matter movement. Just as Bowden and Cullen write: ‘truth be told, having to work harder to obtain similar opportunities to our colleagues takes an emotional toll. We are tired’ (2021, p. 760), Walton and colleagues say: ‘We are sick and tired, of being sick and tired!’ (2021, p. 252).

The impact of COVID-19 on the next generation of Black graduates and the Black staff in Higher Education is thus significant. However, its long-term effects on Black staff and students are unknown (Pennant, 2022) , and there are a number of calls for further research that explores the impact of COVID-19 on Black communities. Indeed, many of the recommendations arising from such research are around engaging in further study (e.g. Phiri et al., 2021), such Proto and Quintana-Domeque (2021) who call for additional research on the potential differential effects of the COVID-19 pandemic by ethnicity. Similarly, the UK Centre for Mental Health highlights a need for research that goes beyond outdated and homogenised comparisons of ‘White’ and ‘BAME’ to focus on specific ethnic groups, without which we cannot truly understand the variances in health and outcomes that emerge between different communities (O’Shea, 2021). Given the paucity of such research within the Academy, it is the aim of the current study to explore the experiences of Black students and staff within the context of the COVID-19 pandemic. Recognising that within the US and British education systems, Black girls and women are often ignored (see Pennant, 2022), this research places particular emphasis on these voices.

### Critical race theory

One way in which to uplift the voices of the Black community is through critical race theory (CRT), a theoretical framework that examines the role of race, racism and power (e.g. Delgado & Stefancic, 2000). With its roots in legal studies and Black feminism (Crenshaw, 1991 & Hill Collins, 2019), a founding tenet of CRT is in its commitment to eliminate all forms of oppression (Dixson & Rousseau, 2005) through an interdisciplinary approach (Solórzano, 1997). Solórzano and Yosso (2002) define critical race theory in education as:

a framework or set of basic insights, perspectives, methods, and pedagogy that seeks to identify, analyse and transform those structural and cultural aspects of education that maintain subordinate and dominant racial positions. (p. 25)

Theoretic elements of CRT include the centrality and intersectionality of race and racism, race as a social construct (Collins, 2004), whiteness as property (Harris, 1995), the challenge to dominant ideology and the value of experiential knowledge (Solórzano, 1997). The first, centrality of race, is about the permanence of racism (Bell, 1992) and how it is so embedded within society that it is difficult to recognise and hard to address (Ladson-Billings & Tate, 1995). It should therefore be foregrounded in research (Solórzano & Yosso, 2002). This includes intersectionality Crenshaw, 1991), acknowledging how the centrality of racism is impacted by other interrelated identities and social structures, such as gender, sexuality and class (Collins, 1998). The second element, social construction, means race is not inherent within itself or a person, but is a complex and longstanding product of society (Collins, 2004). Third, whiteness as property refers to the value whiteness holds in relation to other races (Mensah & Jackson, 2018). Historically, this was in the context of property and rights, but over time has become associated with other abstract concepts such as time, creativity or education (Harris, 1993). The fourth, challenge, means countering dominant narratives around neutrality, objectivity, colour-blindness and meritocracy in society, all of which maintain the value of whiteness. One way in which to challenge these ‘majoritarian stories’^4^ is through a fifth element, experiential knowledge. CRT research values the lived experiences and narratives of minoritised communities, i.e. counter-storytelling (Delgado & Stefancic, 2012), hence its use in the current study.

Utilising a critical race theory (CRT) framework, this paper attempts to explore the disproportionate impact of the COVID-19 pandemic on Black students and academic staff in UK and US higher education. Importantly, this paper will argue that systemic racism within the Academy plays an integral part with regard to reproducing social and health inequalities, particularly in the midst of a global pandemic. This ran concurrently alongside the murder of George Floyd and subsequent global outcry, which was a key topic for discussion. The study emanates from the marginalised voices of forty-three Black students and academic staff within the UK and US higher education sector. Exploring the impact of the pandemic against of backdrop of structural and institutional racism serves as an important vehicle not only for promoting counter-storytelling narratives but also advocating to redistribute the labour of anti-racist endeavour away from Black members of the Academy.

## Methodology

### Participants

The study involved thirteen US-based universities, including Ivy League, R1 and Historically Black Colleges and Universities (HBCU). It also included nine UK-based universities, including Russell Group and Post-1992 institutions. As experiences may differ depending on institution (e.g. HBCU or the predominately White Ivy League), the diversity amongst universities was deemed suitable providing a broad range of perceptions, giving the research a holistic outlook on participant experiences of COVID-19 within the USA and UK. Purposeful sampling was used, and recruitment of participants was facilitated through networks and recommendations among Black staff and students working and studying within the Academy.

The recruited participants (*n* = 43) were Black staff (*n* = 18) and Black students (*n* = 25). The staff, aged between 28 and 60 years old, had also been students in higher education at some point in their lives. Of these, 14 (78%) were female and 4 (32%) were male. The students were aged between 18 and 25 years old and were attending a range of undergraduate, postgraduate taught and postgraduate research courses. Moreover, while both staff and students share experiences of racism in US and UK education, their experiences are multifaceted due to their positioning in the university fitting the scope for becoming the sample of this study.

### Procedure

Participants engaged in individual, semi-structured interviews (*n* = 42) and focus groups (*n* = 8) to explore their lived experiences of navigating the pandemic whilst in higher education. To inform the focus groups, all participants completed a reflective journal to chart and document their experiences of COVID-19. These became reference points and points of departure within the facilitated group discussions.

Participants were contacted through purposeful sampling aided by both researchers institutional and personal affiliation to UK and US institutions. The objectives of the study were explained to all the participants, and informed consent was ethically obtained. Participants were drawn from a combination of staff and students from UK and US higher education institutions. These ranged from the Russell Group^5^ and Post-1992^6^ universities in the UK; and Ivy League,^7^ Historical Black Universities^8^ and R1 universities in the USA.^9^ The sample comprised of eighteen students and twenty-four members of staff at mid-career level. In the total sample of forty-two participants, there were twenty-six Black women (sixteen students and ten staff) and sixteen Black men (seven students and nine staff). All were interviewed on multiple occasions between 2019 and 2021 through a mixture of in-person and online interviews. Discussions were facilitated by the researchers who had experience in cross-cultural working and qualitative methods. All focus group sessions and interviews were audio-recorded and transcribed verbatim, before being coded in NVivo and data analysis applied. Across the 3-year period, a total of eight different focus groups containing a minimum of three participants during each session were conducted.

### Design

The analytic process was driven by a CRT conceptual framework, which informed the development of knowledge constructed through interactions between the researcher and participants. In terms of positionality, the researcher had lived experience both as a Black academic and students within higher education. It is recognised that associated insider/outsider statuses can be both a benefit and a limitation of the research process (Bhopal, 2010), and thus the role of the reflexive researcher (Young, 2004) becomes especially important here. For example, both researchers’ sharing lived experiences of racism could display (i) bias and/or (ii) racialised flashbacks, affecting the procedure stage illustrated in the previous section. It is undeniable researcher bias affects data validity, and thus to limit our biases, the researchers kept their subjectivity to not conform to one truth supporting different perceptions. Additionally, countering racialised flashbacks was considered, i.e. the researchers personal experiences of diverse forms of racism coinciding with participants perceptions during the interviews. Thus, self-reflective notes and joint-researcher communication was used to protect mental health, preventing any responsive emotions during the interview processes.

The interview design was informed by a critical race-grounded methodology process (Malagon et al., 2009) that captured the experiences of Black staff and students during the pandemic, including the concurrent Black Lives Matter movement. A grounded theory rejects the notion of positivist paradigms and methods taking a deductive approach to research creating an ‘apartheid of knowledge’ (Glaser & Strauss, 2017; Malagon et al., 2009), which results in reoccurring narratives of deficit approaches towards people racialised as Black. This is congruent to CRT, where a CRT methodology centres the voices of groups who are racially marginalised to challenge these constructions from traditional orthodoxies aka Eurocentric epistemologies co-constructing racism in the form of White supremacy. In sum, this means while CRT was used conceptually to guide the structure of the interview schedule, the amalgamation of grounded theory was flexible enough to allow participants to divulge their stories.

Another methodological point of significant importance was the fact that both researchers were Black men and some of the key findings in the forthcoming discussions concerned the gendered implications of racism and COVID-19 for Black women in particular. As well as contending with the ways in which lived experiences of racism, and especially anti-Black racism, would be an unavoidable aspect of both the way we conducted the research and our interpretation of the data, it was essential for our CRT framework to reflexively contend with race, gender and power (Hill-Collins, 2015). This required the researchers to, firstly, consider how they engaged with the participants and, secondly, how they interpreted the Black women’s reflections concerning how institutions had disproportionality treated them in comparison to some of the Black men’s narratives. While this paper contributes to scholarly interventions on the particularities of Blackness, racism and COVID-19, the researchers remain cognisant of how the intersection of gender should not be understood as supplementary to the findings, but rather, as an *always already* symptom of what bell Hooks’ (2000) contends as a key pillar of *white supremacist capitalist patriarchy* which infiltrates all forms of racism (and the research we conduct to illuminate these patterns). In this way, as custodians of anti-racist endeavour, it becomes our scholarly duty, as well as being integral to our position as Black men to be conscious that when discussing and researching the treatment of Black women in the workplace, we keep hooks’ note on how ‘interlocking systems of classism, racism, and sexism work to keep women exploited and oppressed’ (Hooks, 2000, p. 109). These intersectional and gendered structures poignantly highlighted by hooks remained that at the forefront of how we engaged with the participants and the data we interpreted on the connection between COVID-19 and racism in universities.

## Findings and discussion

Three themes were identified through analysis. The first theme, pandemic within a pandemic, had two sub-themes around *racial (re)traumatisation* and *loneliness and isolation.* A second theme emerged, job precarity, alongside a third theme, exploitation of Black labour. The key tenet of CRT incorporated in the aforementioned themes is concerned with asserting that racism as ‘ordinary, not aberrational’. Thus though the discussions are located in the context of universities, the analytical framework demonstrates that racism is ‘normal’ and the living everyday experience (Delgado & Stefancic, 2000: xvi). It is our contention through CRT—and connecting individual narrative with the aforementioned global racist structuring of society—that COVID-19 exacerbated all forms of racisms and, in particular, anti-black racism.

### Pandemic within a pandemic

There was a strong theme around racism and COVID-19 representing a ‘pandemic within a pandemic’ for Black people (Laurencin & Walker, 2020). For many participants, it was impossible to separate the impact of anti-Black racism, which was especially illuminated by the Black Lives Matter movement, and the impact of COVID-19, which was disproportionate among Black communities:

The impact has been difficult, as a Black member of faculty my experience has obviously been compounded by the events of George Floyd and BLM and a President^10^ that had no care for Black lives. (P3, Black Female, Staff, US Higher Education)

For participants three and thirteen, both Black US staff, experiences of racism during the pandemic were not only magnified through COVID-19 and the murder of George Floyd, but also within the White House. President Trump compounded the US racial divide, with both participants experiencing an acute disregard for Black lives.

The pandemic was hard. In the US, we were up against it on several fronts: we were being failed terribly by a xenophobic President^5^ who was whipping up as many racial divisions as possible. All of this in the midst of the George Floyd murder and the disregard by the President for his life. (P13 Black Male, Staff, US Higher Education Institution)

Former President Trump was a percolating theme for US participants as it was continuously perceived that his actions played an important role in their experiences during the pandemic. For the US participants, the pandemic was exacerbated by the lack of involvement from the government which clearly had demonstrable effects on their sense of self (subtheme below on loneliness). As the governmental system reverted from a true engagement with anti-racist policies, it instead constructed *racist policies* (e.g. trump banning CRT or anti-racist training in schools and the workplace, see Morgan, 2022). This highlights the systems connectivity with an original CRT doctrine ‘white dominance’ where ‘white supremacy’, i.e. ‘everyday mundane actions and policies that shape the world in the interests of White people’ (Roediger, 2007 & Gillborn, 2018, p. 39) impact communities who are racially marginalised.

Moreover, participant perceptions also illustrate how White dominance creates discrepancies in the Black and White experience during the pandemic (Roediger, 2007). For example, as reflected in the comments from participant nine, for Black people, living in a world designed by and for White communities requires constant negotiation and navigation. While their White counterparts were dealing with one pandemic, Black people were dealing with two:

Negotiating the pandemic has been very difficult. As a Black person it’s highlighted even more the blatant disregard for Black Lives. (P9, Black Female, Student, UK Higher Institution)

Yosso (2005) identifies navigational capital as ethnic minority students’ skills and abilities to traverse social and educational institutions, which are often unsupportive or hostile environments that neglect and were not created with communities of colour in mind. This additional resource, which beneficiaries of ‘white supremacy’ are neither required to develop nor value (Sue, 2006), speaks to the ‘Black tax’ (Harper, 1985) and burdens described both by Walton et al. (2021) and participant eighteen:

During the pandemic seeing the rise of the Black Lives Matter movement globally, it makes you sit and think about the inequality we suffer as people of colour – including Covid. It weighs heavy on you after a while. (P18, Black Male, Staff, UK Higher Education Institution)

This comment reflects wider research naming racism as a co-factor to COVID-19 (Godlee, 2020) and the acute awareness that the Black communities’ experiences have been worse as a result. As well as pointing to the centrality of racism, which for many of the Black participants underscored their experiences of the pandemic, the participant also speaks to a sub-theme regarding the reinforcement of Black suffering, i.e. structured emotional consequences at the hands of racism.

### Racial (re)traumatisation

This sub-theme theme was around the impact of racism on mental health during the pandemic, and specifically how the murder of George Floyd and subsequent Black Lives Matter movement was linked to feelings of trauma and suffering, particularly among student participants. For young Black men, death was represented not just in rising and disproportionate COVID-19 figures (e.g. APM Research Lab, 2021), but in policing, which was the leading cause of death in young Black men in America (Edwards & Esposito, 2019):

A big thing for me during Covid was trying to access mental health support through my university and when I tried to explain the impact that George Floyd murder had on me, like, being a triggering incident for my own personal experiences of racism, they [the counsellors] dismissed it as me being hypersensitive’. (P4, Black Male, Student, US Higher Education)

This participant’s experience is consistent with the findings of a large US study demonstrating that police killings of unarmed Black Americans have adverse effects on mental health among Black Americans (Bor et al., 2018). Their account also reflects existing research highlighting the challenges Black students have accessing mental health services (Arday, 2018). Black men are seen as stronger (Wilson et al., 2017), more impervious to pain (Cintron & Morrison, 2006), and as such are undertreated across both mental and physical health spheres. Both are indicators of racism and patriarchal masculinity (Hooks, 2003) and point to a need for reform within current mental health and pastoral services.

Given research into collective trauma (Eagle, 2014), which the Black community experience both through ongoing racism (e.g. DeGruy, 2005) and the COVID-19 pandemic (Ettman et al., 2020), the experiences of student participants are perhaps unsurprising:

It was painfully obvious to see the disproportionately during Covid…it just speaks to the inequality we face daily. (P7, Black Female, Student, US Higher Education)

My experience has been hugely affected by the pandemic and to be honest the murder of George Floyd. It’s just an everyday reminder of what we have to go through. (P8, Black Female, Student, UK)

It was unclear to me how I would mentally and physically cope with the combination of Covid-19 and racism. It was just constant trauma. (P15, Black female, staff, UK Higher Education)

The above and previous comments suggest the intersections of race and location illustrate the role George Floyd and Black Lives Matter had within the student sample during the pandemic. Despite participants residing in the UK, the George Floyd murder—in the USA—had an impact, and this coincides with P18 above mentioning the role and rise of Black Lives Matter ‘globally’. Thus, racism can be experienced vicariously for Black participants also known as ‘secondhand racism’ (Truong et al., 2016), whereby Black people on the witnessing end (e.g. social media) of racial abuse are impacted by it too.

This reference to the ‘everyday’ and ‘daily’ nature of racism, often referred to as ‘death by a thousand cuts’ (Sue, 2006) , sits alongside how the participants understanding of the murder of George Floyd was a more ‘deeply felt wound in Black communities that extends beyond the individuals that directly experience it…this type of collective trauma must be understood as an urgent public health crisis’ (Waldron, 2021, p.29). These repeated lacerations are linked in the wider literature to racial stress (Carter et al., 2013), racial trauma (Williams et al., 2018) and racial battle fatigue (Smith et al., 2011), especially among African American students in higher education institutions (e.g. Ragland Woods et al., 2021). Ragland Woods and colleagues highlight how these experiences both compound and are compounded by feelings of isolation and disconnectivity, which also emerged as a sub-theme in the current study.

### Loneliness and isolation

A second sub-theme emerged around feelings of loneliness and isolation. This is consistent with wider research that student and ethnic minority groups were at higher risk of being lonely during the UK lockdown, although the latter were already considered at greater risk prior to the pandemic (Bu et al., 2020). Participant five describes the challenge these feelings brought with them:

It was really difficult, I felt a sense of loneliness that was really difficult…and disconnectivity. (P5, Black Male, Staff, UK Higher Education Institution)

For participant nine, this difficulty was directly linked to disconnection from the Black student community:

The pandemic was a very difficult period. I missed the sense of community that being around other Black people. I entered University as a mature student, I’d say this really impacted in terms of my mental health and what I was achieving. (P9, Black Female, Student, UK Higher Institution)

We are communal beings, and a central tenet of Black resilience is in what Yosso (2005) describes as community cultural wealth (p.70). The lack of access to support and community highlighted by participant nine, a Black woman, reaffirms the question asked by Pennant: ‘who’s checkin’ for Black girls and women in the pandemic within a pandemic?’ (2022, p.1). This is important when considering ways in which to increase support for Black students, particularly Black women who typically receive less support from the educational systems around them (e.g. Ricks, 2014). The intersections between race and gender play a pivotal role in the pandemic within a pandemic as the system is especially susceptible to neglecting the lives of Black women.

Having access to supportive relationships not only underpinned participant nine’s mental wellbeing, but also her productivity. This is also echoed in the experience of a Black female staff member:

It gets a bit lonely sometimes. It feels like a lot is on you, you know. It’s a lot of pressure to keep it up…sometimes you feel like you can’t. But you have to, especially in these times, where you don’t know if you’re coming or going. (P10, Black Female, Staff, UK Higher Institution)

Participant ten associates her loneliness with pressure, and a sense that she must keep going regardless. For Black staff, who may not know if they are ‘coming or going’, perhaps due to job precarity or fear of reprisal or exclusion, stopping or slowing down might be seen as privilege not afforded to them. This speaks to the type of loneliness experienced through a lack of care from others, as well as an emergent theme around job precarity and how this may be compounded in ‘these uncertain times’.

The participant testimonies highlight that the pandemic within a pandemic is conditioned and maintained by the structures of whiteness that marginalise the lives of Black people both physically and psychologically. Through the neglect of the health and wellbeing of Black students and staff in higher education, the ongoing backlash at both governmental and institutional level has created a perfect storm of marginality underpinned by a global emergency and the ongoing generation of anti-Black racism (Gillborn, 2018). Therefore, the theme ‘pandemic within a pandemic’ is underpinned by the normalisation of white dominance and anti-Black racism, where the government and higher education institutions (e.g. unsupportive counselling services) reinforce a racist system that creates negative emotional consequences for Black lives (e.g. re-traumatisation and loneliness).

## Precarious employment

Black staff in higher education institutions relayed their anxieties around job insecurity, including the uncertainty around what this could mean for them:

The pandemic has been very difficult. I was on a temporary contract, so my thoughts also gravitated towards the economic impact of Covid and where this would leave me professionally. (P4, Black Male, Staff UK Higher Institution)

Black people in the UK are twice as likely to be employed on zero-hour contracts (UCU, 2021). The associated uncertainty, as described by participant four, feels even more compounded when factoring in national economic crisis and global recession (UK Parliament, 2021). It is unsurprising that job insecurity interrelated with wellbeing, as was the case with a US participant:

I found the pandemic really debilitating, not only did I lose my job but my mental health was also severely impacted. This for me was extremely difficult given how long it had taken me to gain employment in academia. My redundancy was because of the pandemic and I guessed what I noticed at my institution and the sector my generally was that a lot of Black people, people of colour were the ones being made redundant. (P11, Black Mixed-Race Female, Staff, US Higher Education Institution)

Importantly, this suggests that redundancy practices are experienced as a form of institutional racism. This speaks to both the centrality of racism and whiteness as property (Harris, 1985), rendering Black staff feeling more vulnerable and more disposable. In more juxtaposing narratives, participant blamed the pandemic for their adverse circumstances but are absent in blaming their racist institution. Again, an interpretation could be the camouflage of institutional racism and how it can use the pandemic as an excuse to conduct racist practices by threatening their job security. Additionally, and similarly to students, gender discrepancies amongst Black staff were evident. Participant four (a male) fears losing his job, while participant eleven (a female) was made redundant. Here, we play close attention to the tenants of CRT and intersectionality in foregrounding the gendered implications of race and power for Black women in particular (Hill-Collins, 2015). The race-gender element has been shown to have diverse impact amongst Black staff supporting studies and statistics emphasising the difficulties for the roles of Black women in academia (Rollock, 2019). These patterns of oppression reinforce Black people’s having to work ‘twice as hard to get half as far’ (DeSante, 2013), paving the way for the exploitative practices that are central to Black staffs’ experiences within the UK and US Academy (see Arday, forthcoming).

## Exploitation of Black labour

A strong theme that emerged related to participants’ experiences of exploitation, specifically staff:

The impact has been increased workload and for most Black members of staff a feeling of exploited labour during these very difficult times. (P16, Black Male, Staff, UK Higher Education)

Participant sixteen revealed exploitative practices as a shared experience among the Black community, which replicates the findings of a recent UK study (Arday, forthcoming). Experiences of exploitation were well-documented in the literature prior to the pandemic. Tuitt and Stewart (2021) argue that US higher education institutions are designed on the premise of plantation politics and resemble entrenched colonial systems and practices of exploitation and domination.

The exploitation of Black people in the academy is not only violent, it’s normal, to the point that none even questions it’. (P15, Black Female, US Higher Education)

This comment reflects the permanence and normalcy of racism (Bell, 1992) in the US education system, and how little remedial or reparative action is taken. Participant fifteen’s use of the term ‘violence’ also speaks to the significant harm caused by these practices, the cuts, wounds and lacerations discussed earlier taking yet another structural form.

For participants in the present study, their experiences of exploitation are confounded by the pandemic and concurrent Black Lives Matter movement. Where the spotlight on racism following the murder of George Floyd brought with it an increased global awareness of the plight of Black communities, the additional labour required to elevate and respond was outsourced to Black staff in the Academy:

I became the person of colour that was designated to mobilise and complete anything to do with race equality. My labour was not acknowledged and I was positioned as being the person that should lead this without any resource, time or support. (P15, Black Female, US Higher Education)

This comment subtly illuminates an entrenched pattern of white fragility (DiAngelo, 2018), which sits on the other side of the proverbial coin to the notions of Black strength, invulnerability and invincibility described earlier (e.g. Wilson et al., 2017). From these myths arise the expectation that Black people must care for, soothe, and protect White people (Menakem, 2021); historically, this was most visible on the plantations but continues systemically today, as reflected in participant experiences and the wider literature. In essence, to uphold this ideology (‘I need you to take care of me’; Menakem, 2021, p. 97), the burden of racism, and the labour of antiracism, must fall to Black staff:

As a Black academic member of staff, I felt that the burden of the university’s objective to become anti-racist fell squarely upon me. This was not only physically crushing but also emotionally crushing. There was this exploitative expectation that as a Black member of staff I would relish the opportunity to have my workload added to without any concession’. (P13, Black Male, Staff, US Higher Education)

I found this period [pandemic] deeply stressful; the George Floyd murder actually created more work for me as my institution placed a lot of pressure on me as the only Black member of faculty to deliver anti-racist interventions and decolonising things… it was a lot and very stressful. (P2, Black Female, Staff, UK Higher Education)

The experiences of participants thirteen and two highlight the emotional and physical toll of racism, harking back once again to the literature around racial stress and trauma (e.g. Carter et al., 2013). Not mentioned in participants’ comments are the silent beneficiaries, their White colleagues, who remain invisible (Sue, 2006) and thus weightless (McIntosh, 2003).

Within the previous two themes, there is a familiarity amongst Black staff and students which suggest that both precarious employment and the exploitation of Black labour are underpinned by a very particular form of anti-black racism dependant on the structures that allow for race or the racialisation of Blackness to be hidden by Whiteness (Lentin, 2020). It seems pertinent to conceptualise that Whiteness is not solely used to normalise Eurocentric culture and its predominant representation in literature, which camouflages processes of Black exploitation and White passivity. It is therefore important that, as well as counter-storytelling, dominant discourses are challenged in action, including the prioritisation of Black care with a shift in labour away from Black staff and students in the Academy (Arday, 2019).

## Conclusion

Utilising a CRT framework, this study qualitatively explored the impact of the pandemic on Black students and academic staff in UK and US higher education institutions. In doing so, it provides a counter-narrative to dominant racial discourse that platforms the voices of Black people as a distinct group. Firstly, as was evident from the dominant theme, Black communities experienced COVID-19 as a pandemic within a pandemic. Racism, ‘the other pandemic’ (Godlee, 2020), underscored all participants’ experiences, which for US staff and students in particular were compounded by the politics and presidency at the time. The use of students and staff together in the overall discussion and arguments presented a move within sociologies of education in higher education which takes seriously the importance of the academic lifecycle in unison. Significantly, the findings show the correlations and continuation of racism(s) experienced across the academic landscape demonstrating the need for more connections to be made between service users and providers especially concerning global emergencies like COVID-19 and systemic racism.

The concurrent murder of George Floyd, and subsequent Black Lives Matter movement, added to a collective *(re)trauma* experienced by both US and UK participants, many of whom also described feelings of *loneliness and isolation*. Importantly, the results replicate existing findings from before the pandemic surrounding the exploitation of Black labour and precarious employment (e.g. Arday, 2022). These represent longstanding systemic issues within the Academy (e.g. Tuitt & Stewart, 2021) that require urgent redress. These themes and subthemes, which are discussed against the existing research landscape, serve to illuminate some of the structural and societal problems that Black students and academic staff experience unceasingly. Ultimately, as every racial group had adverse experiences under the ‘Same Storm’, i.e. COVID-19, those experiences are diverse requiring different levels of bespoke attention. The juxtaposition of experiences is evident as people racialised as Black remain in *the eye of the storm*, i.e. pandemic within a pandemic, underpinned by anti-black racism and the dominance of Whiteness (Roediger, 2007). The subsequent neglect experienced by Black people aligns with their encounters which metaphorically situate them in stormy terrain. Therefore, purposeful recognition from institutions which take seriously how the inequitable structures of society infiltrate university cultures remains integral if Black people are to be given the space to locate the tools required to navigate the storm.

### Limitations

As with all qualitative research, the findings in the current study are not generalisable; rather, the purpose of this study was to tell the stories of Black students and academic staff navigating the US and UK Academy during the COVID-19 pandemic. One of the benefits of CRT is that these voices, which are often marginalised from discourse or homogenised with other ethnic groups, are centred and brought to the fore. It is recognised that wider perspectives may have further informed this area of research, such as exploring the experiences of Black professional service staff as well as scholars. Conversely, narrower perspectives may also benefit further study, such as more detailed exploration of the experiences across the intersection, such as gender, class or disability.

### Implications and recommendations

For Black students and academic staff, this study points to the complex and intersecting nature of two pandemics, COVID-19 and racism. While the present study initially set out to explore the COVID-19 pandemic, it was impossible to separate participants’ experiences of racism. Together, they continue to reproduce greater inequalities for Black communities, reinforcing the need for each to be considered urgent public health crises. Specifically, both should be recognised as a type of collective, cultural trauma (Waldron, 2021), as reflected in the current study and wider literature and particularly in respect to the murder of George Floyd (Bor et al., 2018).

This recognition must take place at every level, with appropriate fiscal investment to ensure culturally responsive support is available. This might, for example, look like embedding in policy the recognition of racial trauma as a serious life event, as is the case at Goldsmiths, University of London. It could also look like reform within university mental and pastoral care provision, such as improving racial literacy among counsellors or engaging in more robust data monitoring practices. Perhaps also, universities and policymakers might consider abolition of precarious contracts (Arday, forthcoming), or local ringfencing of funding for equality and diversity resources. At the very least, senior leaders should be initiating courageous conversations (e.g. Singleton, 2021), which start from the premise that racism is central and pandemic. Regardless of the approaches taken, there must be a clear shift in where the labour of this work sits, not just formally and in the more observable spaces, but in the quieter (sometimes silent) moments where everyday racism takes place.

## Footnotes

1 Rather, it is assumed.

2 General Certificate of Secondary Education (GCSE), an academic qualification typically undertaken aged 16 years old; and Advanced Level (A-Level), an academic qualification typically undertaken aged 18 years old, both in the UK.

3 The levy imposed on Black people to work harder and, ‘in addition to the usual stresses, confront a set of personalised social strains which grow out of their “Blackness” in predominantly White environments’ (Harper, 1985, p. 207).

4 The majoritarian mindset is defined as ‘the bundle of presuppositions, perceived wisdoms, and shared cultural understandings persons in the dominant race bring to the discussion of race’ (Delgado & Stefancic, 2000, p 462).

5 The Russell Group is a self-selected association of 24 public research universities in the UK. The group is headquartered in London and was established in 1994 to represent its members’ interests principally to Government and Parliament. Representing the UK’s leading universities, the Russell Group has historically been committed to maintaining its status as research intensive in addition to having unrivaled links with business and the public sector (The Russell Group 2018).

6 New University, synonymous with Post-1992 University or modern university, is a former polytechnic or central institution in the UK that was given university status through the Further and Higher Education Act 1992 or an institution that has been granted university status since 1992 without receiving a royal charter. (Armstrong 2008).

7 1 The Ivy League consists of eight private research-intensive universities in the North East of the USA. These universities regularly score highly in league tables based on research and teaching. The Ivy League are the most selective universities in the world (QS World Rankings, 2020).

8 Historically black colleges and universities (HBCUs) are institutions of higher education in the USA that were established before the Civil Rights Act of 1964 with the intention of primarily serving the African-American community.

9 There are 146 institutions that are classified as ‘R1: Doctoral Universities – Very high research activity’ in the Carnegie Classification of Institutions of Higher Education as of the 2021 update.

10 At the time, this was President Trump, who considered himself a nationalist and whose racial sentiments connected to America’s long history of racist stereotypes and discrimination (Clayton et al., 2021).
